# HL-Kids-NRW – Study of fourth-graders’ health literacy

**DOI:** 10.1093/eurpub/ckac129.318

**Published:** 2022-10-25

**Authors:** T Bollweg, O Okan

**Affiliations:** Department of Sport and Health Science, Technical University Munich, Munich, Germany

## Abstract

**Background:**

Although children are identified as a key target group for interventions targeting the development of health literacy, little data is available on children's health literacy and how it is related to health outcomes at a young age. This study addresses this research gap by providing data on fourth-graders’ health literacy and associated health outcomes.

**Methods:**

A cross-sectional study was conducted among fourth-grade students in the state of North-Rhine Westphalia, Germany. The study was designed as a representative survey starting in 12/2020, which could, however, not be realized due to pandemic-related constraints. Data collected between 07/20 and 11/20 is included in the analyses presented here. Among others, we assessed self-reported health literacy with the HLS-Child-Q15 questionnaire, while also assessing various self-reported health outcomes.

**Results:**

n = 364 students are included in the analysis, 49,5% of which are female. The mean age is 9.5 years (SD=.7). The HLS-Child-Q15 demonstrated high internal consistency (Cronbach's α=.812). Self-reported health literacy is high, with a HLS-Child-Q15 mean score of 3.13, indicating that it is rather easy for participants to deal with health-related information. Health literacy is significantly associated with a number of outcomes related to health status (KINDL-R subscales physical wellbeing and mental wellbeing, Spearman's ρ = .280 and ρ = .271, respectively; p < .001) and health behaviour (freq. of brushing teeth: ρ = .173; p < .01; freq. of eating fruit and vegetable: ρ = .217 and ρ = .299; p < .001; freq. of physical activity: ρ = .279; p < .001).

**Conclusions:**

While the overall level of health literacy in our sample is high, higher health literacy is associated with better health behaviours, as well as improved mental and physical wellbeing. Further analyses are necessary to explore the causal pathways between the investigated variables, and representative survey are needed to verify these findings.

